# The Bladder Troubles of Old Men1Read at the 36th annual meeting of the Medical Association of Central New York, held at Auburn, September 22, 1903.

**Published:** 1903-12

**Authors:** William B. Jones

**Affiliations:** Rochester, N.Y.


### The Bladder Troubles of Old Men.[1]

By WILLIAM B. JONES, M. D., Rochester, N. Y.

MEDICAL and surgical science and art that have accomplished such wonderful things for women suffering from diseases peculiar to them, have until recently been unable to do more than palliate certain serious afflictions to which old men alone are liable. Retention of urine and its results are so common that every physician sees a case every little while and if the subject is mentioned in a company of medical men each one can recall cases in his own experience. It is estimated that one out of every ten aged men is afflicted, and of these a large proportion are so much so as to need radical treatment.

There can be no condition more pathetic than that of such a man whose active life is nearly spent, who through it all has anticipated a few years of quiet and comfort to enjoy the companionship of friends and family when he would be unable to work longer for them. Instead, he is tormented by the almost constant necessity of trying to relieve the bladder, accomplished but only partially and unsatisfactorily many times every day and night, and during an aggravation of the condition each effort is attended with an agony of spasm and pain. Worn with suffering the poor old man endures until exhausted he falls into a troubled delirium from which the only relief is death,which comes to him as the greatest possible mercy and to his friends most welcome, ending it all. That is only part of the story. There are the gradual onset and course of disease extending over a few years, the first enfeeblement of urination, when it is tardy and too frequent, because the bladder is not entirely emptied at any time, the first attack of retention after getting cold, wet or over tired, beginning catheter life with its dangers which result in permanent aggravation of the trouble, cystitis, which confirmed catheter users acquire from the use of the instrument, and all who suffer from retention acquire from that cause even without catheterism. There are enough exceptions to the last two statements to prove the rule, but no more.

The only obstructions to be considered are stricture and diseased prostate. Where the latter cannot be discovered and the former exists the appropriate method of urethrotomy should be employed. The external is now so simple and safe that it should be preferred if there can be any doubt about the internal being sufficent. After cutting down upon the membranous urethra it is

1. Read at the 36th annual meeting of the Medical Association of Central New York, held at Auburn, September 22, 1903.

always possible and comparatively easy to pass a sound through the most obstinate stricture, which once done the caliber can always be kept as large as desired. There is no disadvantage in resecting the whole of the urethra for a short distance, because mucous membrane reforms where removed and reëstablishes continuity of epithelial surface. Few strictures are neglected until the complications have brought a man so low, and nearly all patients now under consideration have prostatic deformities as a cause of their illness. I use the words prostatic deformities purposely. We must forget that we have considered this necessarily a hypertrophy, because it is often otherwise. There may be a hypertrophy of the gland as a whole, either symmetrical or irregularly placed.

General hypertrophy obstructs the urethra by compression alone, or if in some part there is a nodule pressing against it it may distort the shape of the canal and flatten it horizontally, vertically, or in any direction. Hypertrophy lengthens the prostatic urethra and necessitates the long, large, curved instrument, the so-called "old man's catheter". It also bulges the floor of the bladder, so as to leave a basin posteriorly which is never voluntarily emptied and raises the urethral inlet so that it is no longer at the most dependent part of the organ. Without the general hypertrophy nodules may grow where they press upon the urethra, distort its shape and prevent satisfactory urination. This will be better understood if one remembers that the tissues of the prostate resemble those of the uterus to some extent and one generally or locally hypertrophied has somewhat the appearance of one with multiple uterine fibroids. They resemble the submucus projecting into the central cavity which is urethral, interstitial, or like the subserous projecting from the outer surface, but it is into the bladder instead of the peritoneal cavity. A superficial nodule is often found to be the only abnormality, being placed just within the bladder at its orifice, blocking the latter, and if the nodule is pedunculated it floats back and forth in the current like seaweed in the tide, but it prevents the passing of any fluid like a ball valve trap.

Many prostates of old men are atrophied, contrary to common opinion, and most of these escape trouble but not all, for with the atrophy there is shrinking which makes the urethra smaller, and the strong sphincter fibers about the neck of the bladder becoming firmly contracted and inelastic form the so-called band of contracture, entirely preventing urination and making the catheter indispensable. The normal prostate lies wholly within reach of the finger when examining by the rectum. Its upper and lateral boundaries can be palpated. General hypertrophy is easily recognised but the other three conditions cannot be. Therefore, if the patient has symptoms of obstruction, he needs treatment for obstruction, whether he has a recognisable general hypertrophy or one of the unrecognisable conditions, localised enlargement along the course of the prostatic urethra, enlarged nodule, whether sessile or pedunculated, that projects within the bladder and obstructs the internal meatus, or a band or contracture at the vesical sphincter, and in each of these last cases the prostate may be of normal size or smaller.

The professiön has not been indifferent to the need of treatment for these men and efforts to palliate and to cure them have resulted in more remedies and operations than could be rehearsed in a short time, but most of them are disappointing and until recently the best were only partially successful. Medication, hygiene and regimen are sufficient for some cases. Those not made safe and comfortable in that way should have an operation. No man who uses a catheter is safe, and if he is compelled to do so surgery should be resorted to.

Of the operations one will answer for one case, another for another, but there is only one that is really the best for all. I refer to perineal prostatectomy as devised by Parker Syms, of New York. Briefly, it consists of an external urethrotomy, opening the urethra in the membranous portion and a little way to the prostatic, the passage of a collapsed rubber ball through this wound along the prostatic urethra into the bladder, to use as a tractor while enucleating the gland or a part of it, if no more is needed. The rubber ball is attached to the end of a soft rubber tube through which water is injected, distending the ball, so that pulling on it may not withdraw it. Traction on the tube now draws down the prostate, making it accessible and easy to operate upon. Also it compresses all tissues of the perineum and thus prevents hemorrhage, which would otherwise be profuse as the manipulation is among a plexus of large venous sinuses. Also there is counter pressure of the rubber ball, against which the surgeon works more rapidly and surely, and a foreign body that warns him of too near approach to the bladder so that he need not tear a hole into it, an accident that would make the final condition of the patient unsatisfactory.

The whole operation is done in five to fifteen minutes. There is no hemorrhage worth mentioning, no shock, and final results are nearly always entirely satisfactory. The patient is in almost the same condition as after external urethrotomy and needs little care, mostly cleanliness. He sits up a little every day after the first and is not debilitated by confinement in bed. During the operation the surgeon is in the best possible position to treat stricture in the urethra or stone in the bladder, the prostate and all the bladder are examined by his finger after excising them, and he can equally well remove one or more nodules alone, the whole prostate, a tumor of the bladder or any condition found. It enables him to treat in the best possible manner every complication and is the only operation that does. It leaves the patient with continuous perfect drainage, a sure prevention of urethra fever, which means septicemia, the best cure for all diseases of the urethra, bladder, ureters and kidneys that have developed as a result of the primary condition.

Finally, there is no mortality except that due to old age, the chances being only very slightly increased by a practically bloodless operation of a few moments duration without shock. If it is delayed until the patients are in the last stages of decrepitude some will fail because of that, but if resorted to while the man is yet in good physical condition there is no unusual danger about it. Everyone should recover and be spared years of misery. Whenever a man has to begin using the catheter the operation should be done, because it should be done when safest and most successful and because it should be used to prevent the local disease, the constitutional consequences, and the general suffering that are impending, rather than later as a forlorn hope to prevent approaching death when it can no longer be delayed.

525 Lake Avenue.

